# Accidentally Discovered Aortic Valve Papillary Fibroelastoma in a Patient Presenting With Inferior Myocardial Infarction

**DOI:** 10.7759/cureus.72783

**Published:** 2024-10-31

**Authors:** Mahmoud A. Ismaiel, Mohamed Salah Shehata, Attaa Khaleel Taha, Abdalla Elagha

**Affiliations:** 1 Cardiovascular Medicine/Cardiology, Global Medical City Hospital, Cairo, EGY; 2 Cardiology, College of Medicine, Kasr Alainy University, Cairo, EGY; 3 Cardiovascular Medicine, University of Baghdad, Baghdad, IRQ; 4 Cardiovascular Medicine/Cardiology, College of Medicine, Kasr Alainy University, Cairo, EGY

**Keywords:** cardiac magnetic resonance (cmr), cardiac papillary fibroelastoma, computed tomographic coronary angiography (ctca), st-elevation myocardial infarction (stemi), transthoracic echocardiography (tte)

## Abstract

Papillary fibroelastomas (PFEs) are rare, benign primary cardiac tumors. Their clinical manifestations range from asymptomatic findings to serious embolic events, including cerebrovascular accidents, myocardial infarctions, or arrhythmias. Though extremely rare, PFEs have been associated with acute coronary syndrome in only a few cases documented in medical literature.

We present the case of a 39-year-old male who presented with acute-onset severe chest pain and was diagnosed with inferior myocardial infarction. Coronary angiography revealed a high thrombus burden in the right coronary artery. Multimodality imaging, including transthoracic echocardiography and cardiac magnetic resonance imaging, identified a well-defined, mobile mass attached to the right coronary cusp of the aortic valve, highly suggestive of a PFE. Following a multidisciplinary consultation, we performed surgical excision of the mass, and histopathology confirmed the diagnosis of PFE. The patient made an uneventful recovery postoperatively.

This case highlights the rare association of a PFE with acute coronary syndrome, emphasizing the importance of multimodality imaging in the accurate diagnosis of cardiac masses. Given the potential for serious embolic complications, prompt surgical intervention remains the treatment of choice in symptomatic cases. Further studies are needed to establish clear guidelines for the management of asymptomatic fibroelastomas.

## Introduction

Among primary cardiac tumors, nearly 75% are non-malignant, with myxomas accounting for approximately 30% of cases in the general population and up to 50% in adults [1]. Papillary fibroelastomas (PFEs) and lipomas are the next most frequently encountered primary cardiac neoplasms. PFEs are rare benign tumors of the heart, typically arising from valvular endocardial tissue. Despite their rarity, they represent the most common tumors of the cardiac valves, especially affecting the aortic and mitral valves [2].

PFEs, mostly asymptomatic, pose a unique clinical challenge due to their potential to cause severe complications. Their small size, often less than 1 cm, may obscure diagnosis, but their high mobility and friable nature increase the risk of embolization. This can result in life-threatening consequences such as stroke, transient ischemic attacks, myocardial infarctions, and peripheral embolization [3]. The risk of such embolic events is particularly high when the tumor's location is on the left side of the heart, where it can easily dislodge and enter systemic circulation. Some cases implicate PFEs in coronary artery obstruction, leading to acute coronary syndromes, a rare but documented presentation in the literature [4].

Given the potential severity of complications, early detection of PFEs is crucial. Transthoracic echocardiography (TTE) and transesophageal echocardiography are the primary imaging modalities for diagnosis, allowing for detailed visualization of the tumor's morphology, attachment site, and mobility [5]. Advances in cardiac magnetic resonance (CMR) imaging and computed tomography (CT) have further enhanced diagnostic accuracy, providing additional tissue characterization and helping to differentiate fibroelastomas from other cardiac masses such as myxomas, thrombi, and vegetations. However, the definitive diagnosis still relies on histopathological confirmation following surgical excision [6].

We present a case of a male patient who initially presented with an inferior myocardial infarction and an accidentally discovered amass attached to the aortic valve, which was confirmed as a PFE by histopathology.

## Case presentation

We present the case of a 39-year-old male patient who presented to the ER on day one with a two-hour history of typical chest pain. He is a non-smoker with no past cardiac or medical history and no family history of cardiac diseases.

**Figure 1:**
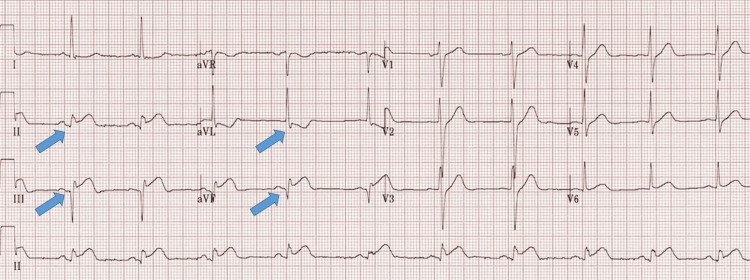
12 lead resting ECG showing ST segment elevation in leads II, III, and aVF with ST-segment depression in lead aVL

An electrocardiogram (ECG) was performed immediately, revealing ST-segment elevation in leads II, III, and AVF, with reciprocal ST depression in leads I and aVL (Figure 1). The patient was loaded with 300 mg of acetylsalicylic acid (ASA) and 180 mg of ticagrelor and promptly transferred to the primary catheterization unit.

**Figure 2:**
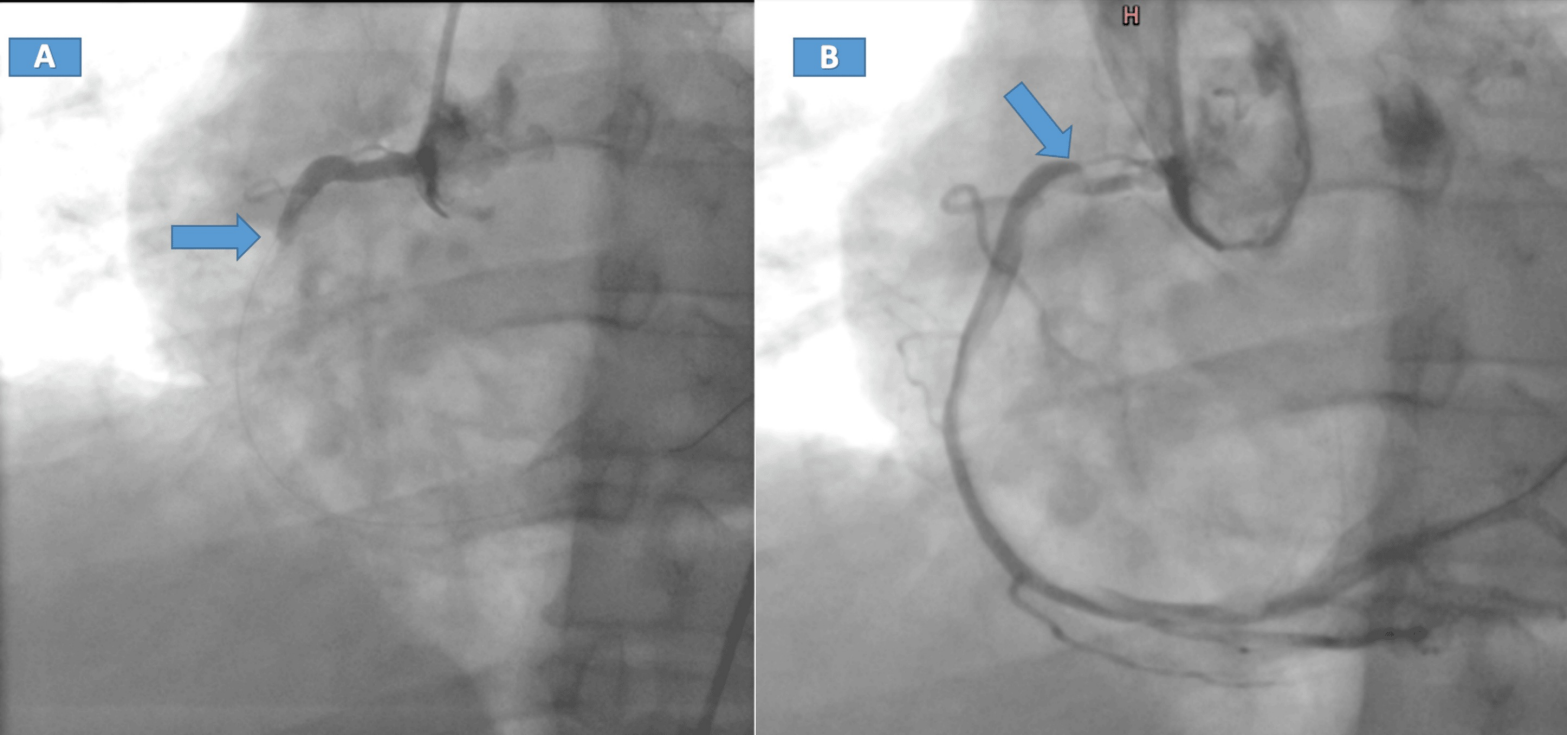
CA showing total proximal occlusion of the RCA (A) and thrombus (B) CA: Coronary angiography; RCA: right coronary artery

**Figure 3:**
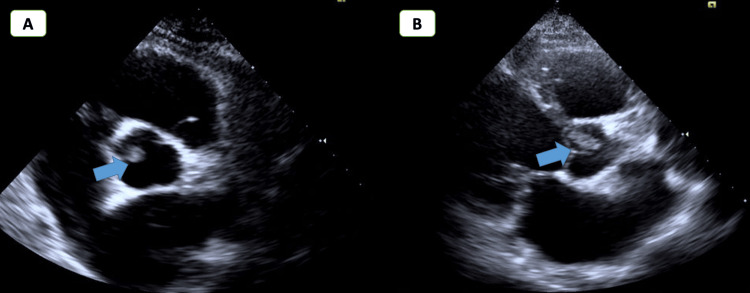
TTE with short and long parasternal axis view showing a mass attached to the aortic valve TTE: Transthoracic echocardiography

The clinical examination was unremarkable. His blood pressure was 100/60 mmHg, and his heart rate was 90 bpm. Coronary angiography (CA) was performed, revealing a high thrombus burden in the right coronary artery (RCA) (Figure 2). After a trial of using aspiration thrombectomy and intracoronary lytic therapy, the thrombus burden decreased with no stenting; the decision here was to complete tirofiban for 18 hours. Following CA, a bedside echocardiogram showed a homogeneous round mass, approximately 1.5 x 1 cm, attached to the right coronary cusp of the aortic valve (Figure 3), with no aortic regurgitation or stenosis, normal left ventricular function, and grade I mitral regurgitation. To further assess the mass and the viability of the myocardium, CMR imaging was requested.

**Figure 4:**
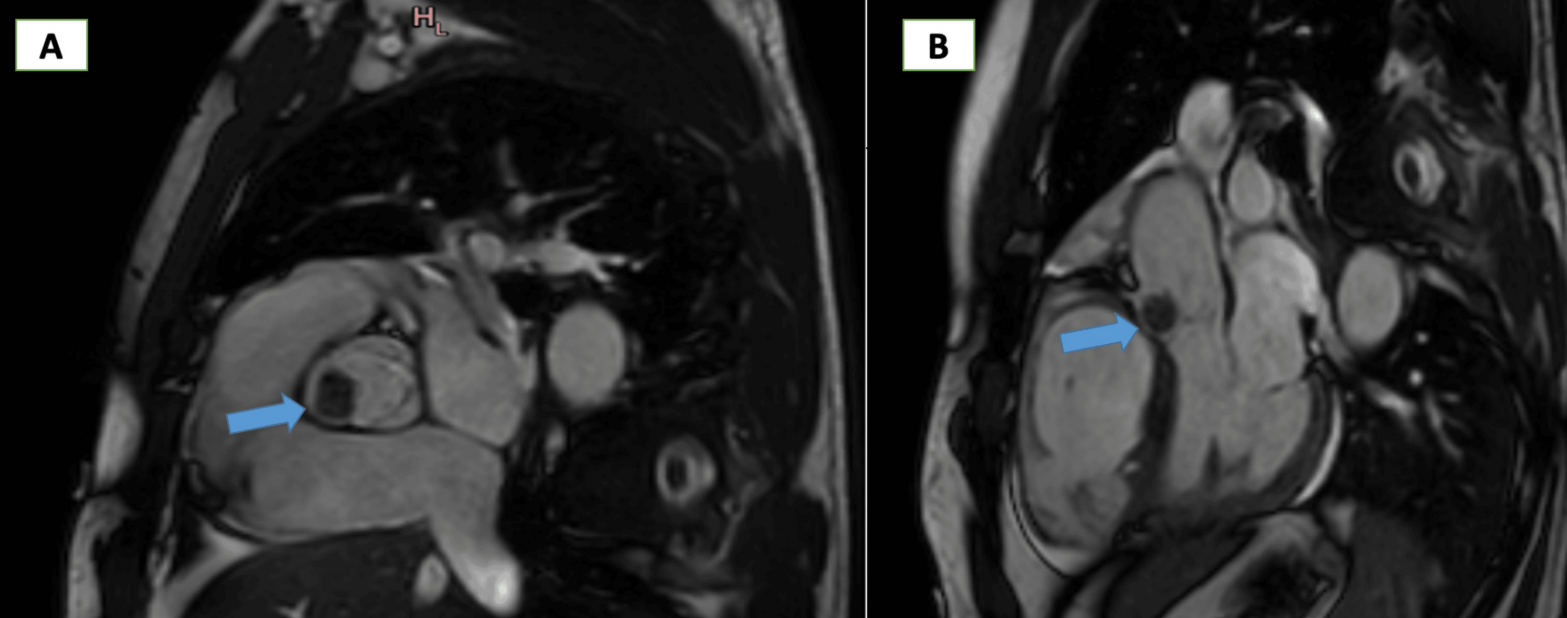
CMR, steady-state free precession image showing a mass attached to the right coronary cusp of the aortic valve CMR: Cardiac magnetic resonance

**Figure 5:**
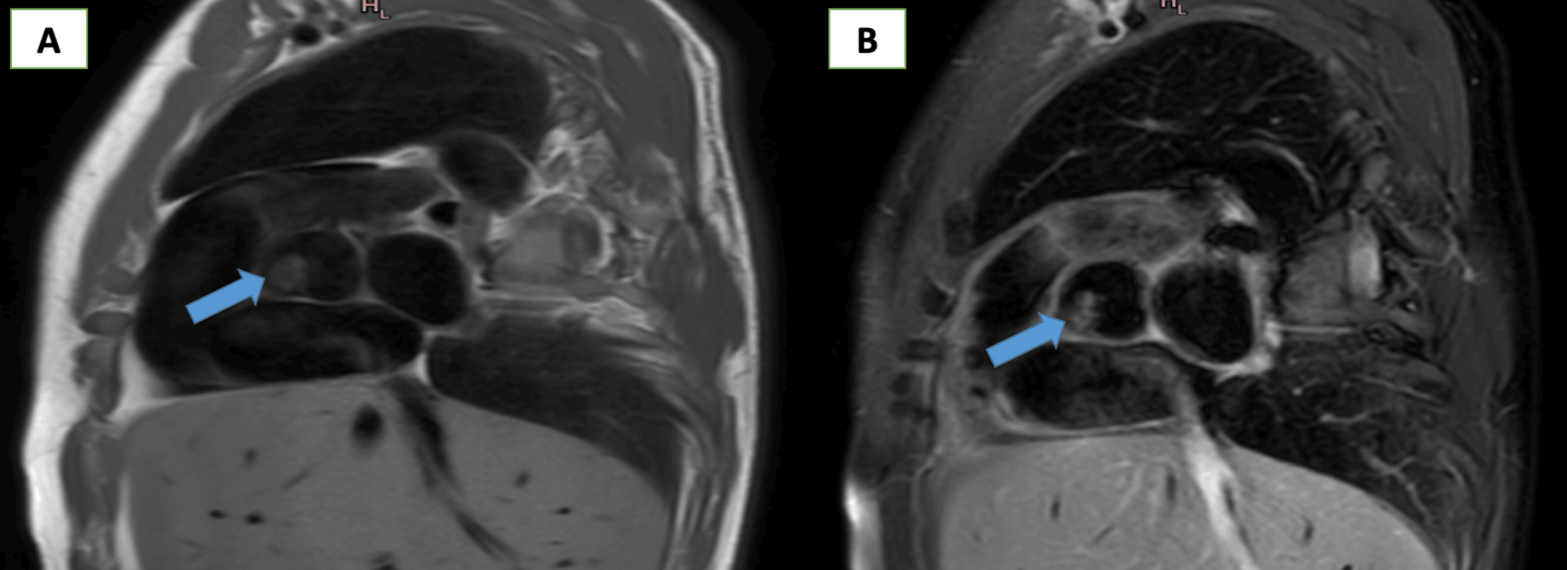
CMR images (A) showing an iso-intense signal of the mass in the T1-weighted image and (B) showing a hyper-intense signal of the mass in the T2-weighted image

**Figure 6:**
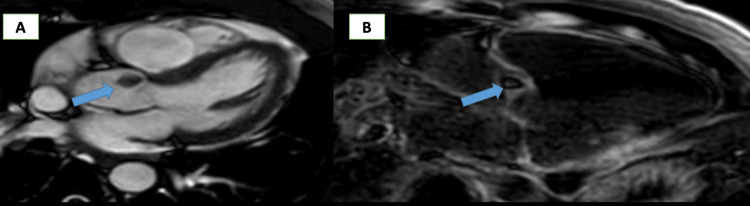
CMR image showing delayed hyperenhancement. The mass shows significant hyperenhancement appearance in a significant part of the mass

CMR was performed on the second day and revealed a small, nearly rounded mass with smooth borders attached to the arterial surface of the aortic valve (right coronary cusp). The mass measured 1.4 x 1.2 cm, was highly mobile, and prolapsed into the left ventricle during diastole. There was no invasion of adjacent structures or obstruction of the mitral valve (Figure 4). On T1-weighted (T1W) images, the mass appeared iso-intense, while on T2-weighted (T2W) images, it was hyperintense (Figure 5). No signal drop was noted on fat-saturation images. On perfusion images, the mass was partially enhanced, and on delayed hyperenhancement (DHE) images, a significant portion of the mass showed hyperenhancement (Figure 6). There were no signs of infection; the autoimmune profile was negative. Along with CMR data, this excludes infective endocarditis and non-infective endocarditis (Libman sack). All these data suggested a benign tumor. The most probable diagnosis was a fibroelastoma for histopathology.

After consultation with the cardiothoracic team, the decision was made to proceed with surgical excision of the mass due to its mobility and embolic manifestation risk. Ticagrelor was stopped, and the patient was observed for five days, then valve-sparing mass excision surgery was performed, and the mass was sent for histopathological examination. Histopathology confirmed the diagnosis of a fibroelastoma.

## Discussion

In this report, we describe a rare case of a patient presenting with inferior myocardial infarction, who was accidentally discovered to have an aortic valve mass attached to the right coronary cusp, which was confirmed to be a PFE by histopathology, which is highly mobile and carries a risk for distal embolism. CMR imaging provides useful information for characterizing cardiac masses by using different sequences (CINE images, tissue characterization, and gadolinium sequences) and offering accurate measures. In our case, CMR excludes thrombus because it is not enhanced in late gadolinium enhancement. The tissue characterization (T1 iso-intense and T2 hyper-intense) strongly suggests the presence of a fibroelastoma.

Primary cardiac tumors are exceedingly rare, with PFEs being the second most common type following myxoma, accounting for approximately 10% of all benign cardiac neoplasms [7]. They are the most prevalent primary tumors affecting heart valves, predominantly involving the aortic valve, with the mitral, tricuspid, and pulmonary valves affected less frequently [8]. While typically asymptomatic and often discovered incidentally, some cases present with serious embolic events, such as stroke, acute coronary syndrome, or myocardial infarction, potentially leading to fatal coronary ostial obstruction [9]. These complications may arise from the embolization of thrombi formed on the tumor’s surface or fragments of the tumor itself.

Over 80% of PFE cases involve the left-sided heart valves, with the aortic valve being the most frequently affected, followed by the mitral valve. On echocardiographic imaging, these tumors may appear as irregular, threadlike, or finger-like structures, often exhibiting branching or flower-like fronds and displaying significant mobility [10]. Fibroelastomas are papillary growths of the endocardium, typically appearing larger and more proliferative than Lambl’s excrescences, which are degenerative in nature. Studies indicate that fibroelastomas exceeding 1 cm in size carry an elevated risk of embolization, warranting surgical excision either as a primary or secondary preventive measure while preserving the integrity of the underlying valve [8].

Echocardiography serves as an essential first-line imaging technique, offering significant advantages in evaluating valve morphology, detecting dysfunction, and identifying cardiac tumors [11]. CMR further enhances diagnostic accuracy [12]. In managing PFEs, oral anticoagulation may be considered a therapeutic option for select cases. However, surgical intervention remains the most effective approach, particularly for primary cardiac tumors that are non-mobile and small (<1 cm) [13]. Surgery is typically recommended promptly, especially in symptomatic patients, due to the increased embolization risk associated with tumors exceeding 1 cm in size [12]. Surgical resection provides excellent outcomes, as complete removal of the fibroelastoma is curative, despite the limited understanding of the condition’s natural progression. According to the literature, the recurrence rate is remarkably low, reported at less than 2% [12,13].

The management of PFEs is influenced by several factors, including the tumor size, location, and symptoms. Surgical resection is generally recommended for symptomatic patients or tumors larger than 1 cm due to the increased embolic risk. Valve-sparing techniques are often employed, given the benign nature of the tumor, with excellent long-term outcomes and a low recurrence rate. For asymptomatic patients with small, non-mobile tumors, close monitoring and follow-up may be an option, though the risk of sudden embolic events remains a concern in such cases [2].

## Conclusions

This case highlights the rare but significant association between PFEs and acute coronary syndrome, underscoring the importance of considering cardiac tumors as a potential underlying cause in patients presenting with myocardial infarction without traditional risk factors. Multimodality imaging, particularly echocardiography and cardiac magnetic resonance, plays a crucial role in the accurate diagnosis and characterization of such tumors. While PFEs are typically benign and asymptomatic, their potential for serious embolic events, including myocardial infarction, necessitates prompt surgical intervention in symptomatic patients. Surgical excision of the tumor remains the definitive treatment, offering excellent prognosis with low recurrence rates. This case reinforces the need for heightened clinical awareness and timely management to prevent life-threatening complications associated with these rare cardiac tumors. With high clinical suspicion, prompt TTE prior to STEMI intervention, without delayed intervention, if available, may help avoid the stenting strategies and downstream complications from that. 

## References

[1] Bussani R, Castrichini M, Restivo L (2020). Cardiac tumors: diagnosis, prognosis, and treatment. Curr Cardiol Rep.

[2] Zoltowska DM, Sadic E, Becoats K, Ghetiya S, Ali AA, Sattiraju S, Missov E (2021). Cardiac papillary fibroelastoma. J Geriatr Cardiol.

[3] Ahmed R, Moaddab A, Graham-Hill S (2022). A case of papillary fibroelastoma of the aortic valve causing an embolic ischemic stroke. Cureus.

[4] Raheela F, Talpur AS, Rasmussen M (2023). Cardiac papillary fibroelastoma: a rare cause of ST-segment elevation myocardial infarction: a case report. Ann Med Surg (Lond).

[5] Lak HM, Kerndt CC, Unai S, Maroo A (2020). Cardiac papillary fibroelastoma originating from the coumadin ridge and review of literature. BMJ Case Rep.

[6] Aggeli C, Dimitroglou Y, Raftopoulos L (2020). Cardiac masses: the role of cardiovascular imaging in the differential diagnosis. Diagnostics (Basel).

[7] Ngaage DL, Mullany CJ, Daly RC (2005). Surgical treatment of cardiac papillary fibroelastoma: a single center experience with eighty-eight patients. Ann Thorac Surg.

[8] Grinda JM, Couetil JP, Chauvaud S (1999). Cardiac valve papillary fibroelastoma: surgical excision for revealed or potential embolization. J Thorac Cardiovasc Surg.

[9] Sun JP, Asher CR, Yang XS (2001). Clinical and echocardiographic characteristics of papillary fibroelastomas: a retrospective and prospective study in 162 patients. Circulation.

[10] Lembcke A, Meyer R, Kivelitz D, Thiele H, Barho C, Albes JM, Hotz H (2007). Images in cardiovascular medicine. Papillary fibroelastoma of the aortic valve: appearance in 64-slice spiral computed tomography, magnetic resonance imaging, and echocardiography. Circulation.

[11] Gowda RM, Khan IA, Nair CK, Mehta NJ, Vasavada BC, Sacchi TJ (2003). Cardiac papillary fibroelastoma: a comprehensive analysis of 725 cases. Am Heart J.

[12] Ikegami H, Andrei AC, Li Z, McCarthy PM, Malaisrie SC (2015). Papillary fibroelastoma of the aortic valve: analysis of 21 cases, including a presentation with cardiac arrest. Tex Heart Inst J.

[13] Makani S, Haoudar A, Al Bouzidi A, Elkettani C, Houssa MA (2022). Papillary fibroelastoma revealed by an acute coronary syndrome with transient ST segment elevation: a case report. Pan Afr Med J.

